# Normothermic Machine Perfusion Reconstitutes Porcine Kidney Tissue Metabolism But Induces an Inflammatory Response, Which Is Reduced by Complement C5 Inhibition

**DOI:** 10.3389/ti.2024.13348

**Published:** 2024-11-13

**Authors:** Eline de Boer, Marina Sokolova, Neeltina M. Jager, Camilla Schjalm, Marc G. Weiss, Olav M. Liavåg, Hanno Maassen, Harry van Goor, Ebbe Billmann Thorgersen, Kristin Pettersen, Dorte Christiansen, Judith Krey Ludviksen, Bente Jespersen, Tom E. Mollnes, Henri G. D. Leuvenink, Søren E. Pischke

**Affiliations:** ^1^ Department of Immunology, Oslo University Hospital, Oslo, Norway; ^2^ Institute of Clinical Medicine, University of Oslo, Oslo, Norway; ^3^ Department of Surgery, Division of Organ Donation and Transplantation, University Medical Center Groningen, Groningen, Netherlands; ^4^ Department of Medicine and Nephrology, Aarhus University Hospital, Aarhus, Denmark; ^5^ Section for Transplantation Surgery, Department of Transplantation Medicine, Oslo University Hospital, Oslo, Norway; ^6^ Department of Pathology and Medical Biology, University Medical Center Groningen (UMCG), University of Groningen, Groningen, Netherlands; ^7^ Department of Gastroenterological Surgery, Oslo University Hospital the Radium Hospital, Oslo, Norway; ^8^ Research Laboratory, Nordland Hospital, Bodø, Norway; ^9^ Center of Molecular Inflammation Research, Norwegian University of Science and Technology, Trondheim, Norway; ^10^ Division of Emergencies and Critical Care, Oslo University Hospital Rikshospitalet, Oslo, Norway

**Keywords:** normothermic machine perfusion, ischemia-reperfusion injury, renal metabolism, microdialysis, inflammation

## Abstract

Normothermic machine perfusion (NMP) is a clinical strategy to reduce renal ischemia-reperfusion injury (IRI). Optimal NMP should restore metabolism and minimize IRI induced inflammatory responses. Microdialysis was used to evaluate renal metabolism. This study aimed to assess the effect of complement inhibition on NMP induced inflammatory responses. Twenty-two pig kidneys underwent 18 h of static cold storage (SCS) followed by 4 h of NMP using a closed-circuit system. Kidneys were randomized to receive a C5-inhibitor or placebo during SCS and NMP. Perfusion resulted in rapidly stabilized renal flow, low renal resistance, and urine production. During SCS, tissue microdialysate levels of glucose and pyruvate decreased significantly, whereas glycerol increased (p < 0.001). In the first hour of NMP, glucose and pyruvate increased while glycerol decreased (p < 0.001). After 4 h, all metabolites had returned to baseline. Inflammatory markers C3a, soluble C5b-9, TNF, IL-6, IL-1β, IL-8, and IL-10 increased significantly during NMP in perfusate and kidney tissue. C5-inhibition significantly decreased perfusate and urine soluble C5b-9 (*p* < 0.001; *p* = 0.002, respectively), and tissue IL-1β (*p* = 0.049), but did not alter other inflammatory markers. Microdialysis can accurately monitor the effect of NMP on renal metabolism. Closed-circuit NMP induces inflammation, which appeared partly complement-mediated. Targeting additional immune inhibitors should be the next step.

## Graphical Abstract

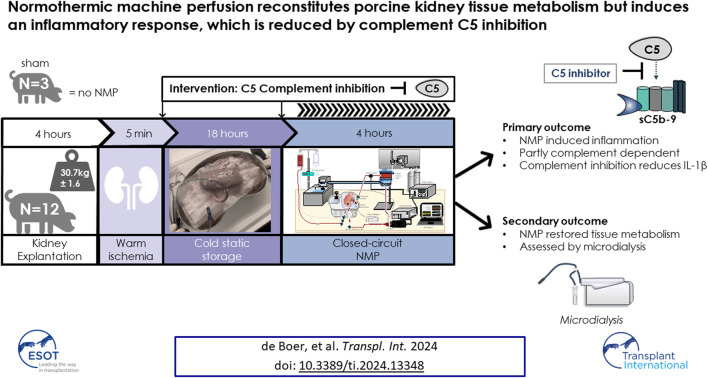

## Introduction

The global shortage of suitable donor kidneys necessitates transplant centers to accept suboptimal allografts which are more susceptible to ischemia-reperfusion injury (IRI) [1–3]. As a consequence, a rise in incidence of clinical manifestations of IRI such as delayed graft function (DGF), primary nonfunction and rejection has been observed [3–5].

Normothermic machine perfusion (NMP) is a promising safe and feasible *ex situ* machine perfusion technique [6, 7]. NMP may alter ischemia and reperfusion induced IRI. During NMP, nutrients and oxygen are delivered to the graft, allowing the continuation of cellular metabolism under near-physiological conditions [8, 9]. Proof-of-concept studies using short-term NMP in human kidney transplantations demonstrated its potential to substantially mitigate IRI [10–12]. Additionally, NMP could be used as a research platform to evaluate non-systemic drug treatment. Yet, NMP itself might lead to inflammation and possible injury, and reliable monitoring tools to track *ex situ* renal metabolic tissue changes are absent.

The hallmark of ischemic injury includes a switch to anaerobic glycolysis leading to increased local accumulation of toxic metabolites [13]. Early detection of anaerobic metabolism during reperfusion is crucial for enabling interventions to optimize compromised grafts. Currently, there are no standard renal metabolic evaluation guidelines, most NMP protocols include estimations of the respiration status based on renal in- and effluent calculations including perfusion dynamics, oxygen- or glucose consumption, final glycolysis products, adenosine triphosphate depletion or focus on mitochondrial evaluation by measuring flavin mononucleotide [14, 15]. Real-time *in vivo* metabolic monitoring of renal metabolism is warranted as it offers the potential to improve nephron viability during NMP. Although invasive, microdialysis is safe, clinically approved, and importantly allows detection of reliable time-dependent metabolic changes in the renal interstitial fluid by using a small probe placed in the renal cortex [16, 17]. Studies on microdialysis in renal grafts, have revealed time-dependent increases in glycerol levels during static cold storage (SCS), and increases in pyruvate levels during hypothermic machine perfusion (HMP). None of the studies on microdialysis have evaluated kidney metabolism during NMP [18–20].

Despite the promising results of NMP in organ preservation, little is known about the inflammatory effect of NMP itself, which might add to tissue damage [21]. The complement system is central in the innate inflammatory response and can be rapidly activated upon contact with foreign (bio)material, damaged cellular components, and blood-gas interfaces, all present during NMP [22–25]. Furthermore, various studies using animal models have demonstrated that complement activation plays an important role as a mediator of kidney IRI [26, 27]. Pharmaceutical targeting of the central complement component C5 seems promising, since C5aR1 and C6 blockade has been shown to ameliorate IRI in mice models [28, 29].

This study evaluated the feasibility of microdialysis to monitor renal cellular metabolism during NMP. The primary aim was to investigate the impact of complement C5 inhibition on renal inflammation during preservation.

## Materials and Methods

### Animals

A total of 15 healthy Norwegian Landrace pigs (*Sus scrofa domesticus*), aged 6 months (30.7 ± 1.6 kg) of either sex were used. Exclusion criteria were: (i) haemoglobin <5 g/dL, (ii) SaO2 < 90% while receiving conventional (0.3) FiO2, (iii) mean arterial pressure (MAP) < 50 mmHg and/or heart rate >150 bpm before cross-clamping of the aorta, and (iv) death before kidney retrieval. The day before the experiment the pigs were housed in the animal facility and provided food and water *ad libitum*. All experiments were conducted by certified researchers in concordance with the European Ethical Guidelines for Use of Experimental Animals and the study was approved by the Norwegian Food Safety Authority (Ref. number: 20/78106).

### Surgical Procedure

Anaesthesia was induced with intramuscular ketamine (60 mg/kg), atropine (1 mg), and droperidol (0.6 mg/kg). Pentobarbital sodium (25 mg) bolus injections were administered if needed for sedation and analgesia was provided using morphine (bolus and continuous infusion 1 mg/kg/hour) until no reaction to sharp hoof-pinching was elicited. After tracheostomy, controlled mechanical ventilation (flow 3 L/min, TV 10 mL/kg, RR 18/min, PEEP 5 cm H_2_O, FiO_2_ 30%) was established and anaesthesia was maintained by 1% isoflurane. An indwelling urinary catheter, arterial pressure monitoring, and a central venous catheter were inserted. Once both kidneys and their vessels were isolated, two microdialysis catheters (CMA 71, 100-kDa pore size, length of 30mm, M Dialysis AB, Stockholm, Sweden) were inserted superficially into the lateral renal cortex using a splitable introducer. These catheters were perfused with Hydroxy-ethyl-starch 130/0.4 (Voluven^®^, Fresenius Kabi, India) through microinjection pumps (CMA 107, M Dialysis AB) at a velocity of 1 μL/min. After 1 h stabilization, sodium heparin (10.500 IE) was given, whole blood was collected and the aorta cross clamped prior to kidney retrieval. Both kidneys endured *in situ* warm ischemia time when systemic blood pressure dropped <50 mmHg of approximately 10–15 min. Once retrieved, the kidneys were immediately flushed with ice-cold Ringer’s acetate at low pressure through a Lifeport cannula (Organ Recovery Systems, Itasca, IL) inserted in the renal artery until the effluent was clear from residual blood. Ureters were cannulated with a neonatal feeding catheter (8Fr). Kidneys from each animal were flushed with preservation solution (KPS)-1 (Organ Recovery Systems). Animals were sacrificed by an intravenous injection of 500 mg pentobarbital, 30 mg morphine, and 50 mmol potassium chloride. Ethylenediaminetetraacetic acid (EDTA; 0.5 M) blood samples were collected throughout surgery, centrifuged at 3000 *g* for 15 min at 4°C and stored at −80°C until further analysis.

### Normothermic Machine Perfusion

**FIGURE 1:**
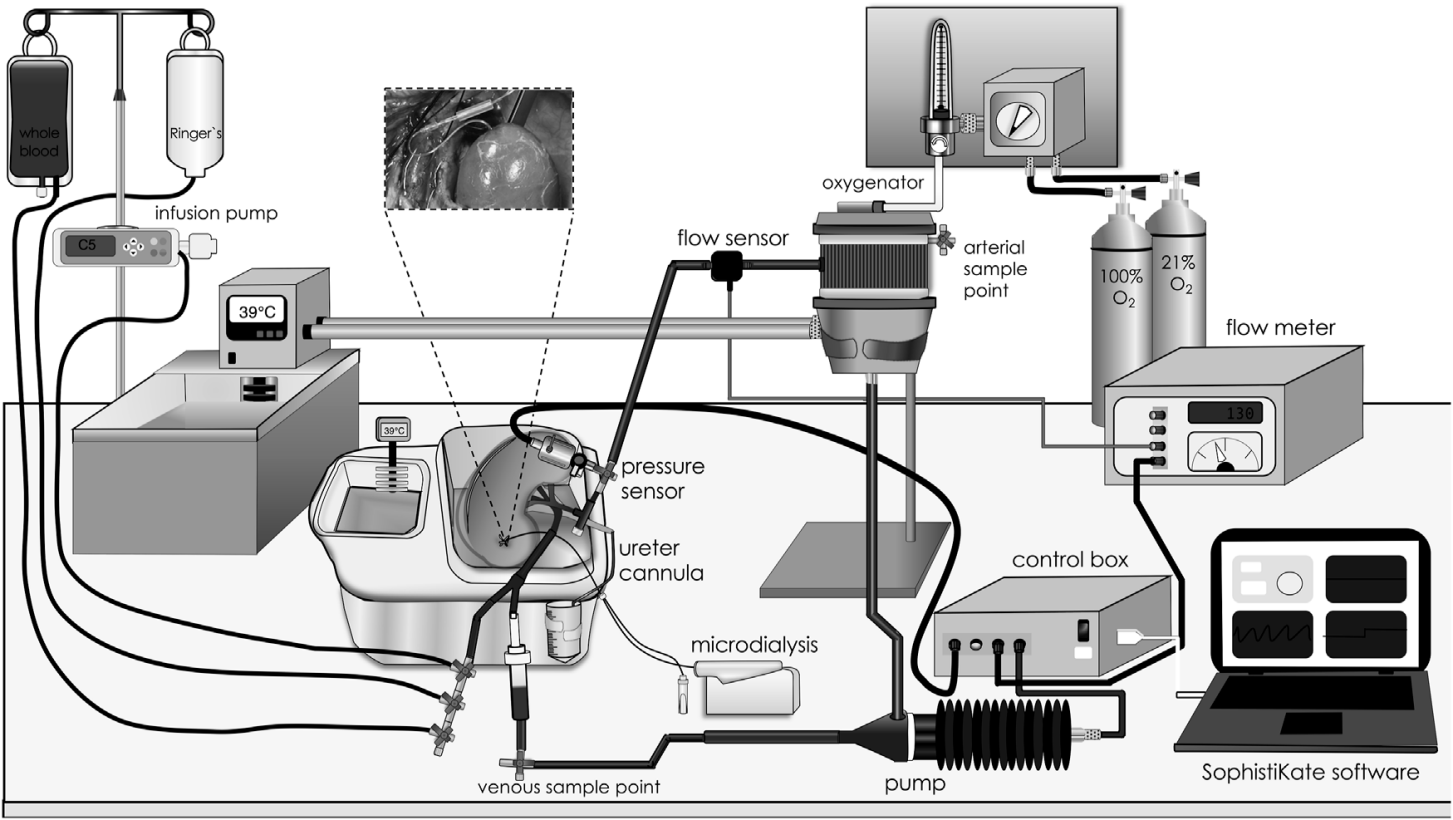
Closed-circuit normothermic machine perfusion. Graphic illustration of the different components of the closed-circuit normothermic machine perfusion model. Blood was driven in a sinusoidal manner at a fixed rate of 60 oscillations per minute by a centrifugal pump. Pump speed was adjusted by setting a mean arterial pressure target in the software. Blood was warmed to 39°C, oxygenated, and cleared for CO2 in an oxygenator prior to entering the kidney through an arterial cannula. At the venous side, treatment, whole blood, Ringer’s solution and urine were infused and pushed into a bubble trap to prevent perfusion of air bubbles from entering the circuit. Perfusate sampling ports were placed before and after the kidney chamber, and the microdialysis syringe pump was placed beside the organ chamber, allowing sampling throughout the perfusion period.

All kidneys were preserved at 4°C for 18 h in University of Wisconsin based preservation solution (KPS-1, Organ recovery systems, Itasca, IL) prior to NMP; sham kidneys were not perfused (n = 6). Closed-circuit NMP was initiated using a pressure-controlled perfusion system (Software: SophistiKate, UMCG, Groningen, the Netherlands), a centrifugal pump providing pulsatile flow (Medos Deltastream DP2; Xenious AG, Heilbronn, Germany), a pediatric oxygenator with integrated heat exchanger (D100: Sorin Group, Arvada, CO) and an organ chamber (Figure 1) [30]. Components were connected by phosphorylcholine coated tubes, sampling ports were situated before and after the organ chamber. Perfusion pressure was obtained via pressure transducers (Edwards Lifesciences, Irvine, CA), and perfusion flow was measured via inline flow sensors (Transonic Systems Europe BV, Elsloo, the Netherlands). The NMP circuit was primed for 20 min with autologous plasma at 39°C (centrifuged at 3000 *g* for 15 min at 4°C). The renal vein was cannulated (12Fr catheter; Sorin Group). NMP was started with oxygenated (atmospheric air/oxygen 70%/30%) whole blood [hematocrit 20%, glucose 1 mg/mL, heparin 5 IU/mL, creatinine 1 mM, 0.1 mL sodium nitroprusside (25 mg/mL: Hospira Inc., Lake Forest, IL)] at 39°C with a mean arterial pressure of 60 mmHg and conducted for 4 h. Volume loss due to urine production was managed by 1:1 volume replacement with the recirculation of urine, administration of Ringer´s acetate, or autologous whole blood in 20 mL intervals based on the blood gas results. Throughout the perfusion, urine and perfusate-preparation samples were collected in EDTA tubes and stored at −80°C. Blood gas analyses were performed (ABL90 Flex/Plus: Bergman Diagnostika, Kjeller, Norway), and electrolyte imbalances were corrected to regulate the pH value. After 4 h of NMP, kidneys were flushed with 200 mL NaCl 0.9% at room temperature and thereafter tissue biopsies (cortex and calyx) were excised and fixed in formalin or snap-frozen at −80°C. Perfusion characteristics including renal blood flow, renal resistance, mean arterial pressure and urine production were constantly monitored.

### C5 Inhibitor

Kidneys from each animal were randomized to receive either 20 μg/mL C5 inhibitor [Ra101295 peptic C5 inhibitor, comparable mode of action to Zilucoplan^®^, provided by Ra Pharma part of UCB Pharma (Brussels, Belgium)] or saline (NaCl 0.9%), thus every animal was its own control. C5 inhibitor or saline was given during SCS (20 μg/mL), as bolus at the start of NMP (20 μg/mL) and as a continuous infusion for the whole study period (1.75 μg/h, Supplementary Figure S1).

### Microdialysis

The microdialysis samples were collected in microvials (M Dialysis AB) during surgery (before and after warm ischemia), SCS (1 h, 3 h, 16 h, and 18 h) and reperfusion (10 min, 30 min, 60 min, 120 min, 180 min, and 240 min). Concentrations of glucose, pyruvate and glycerol were immediately analyzed with the Iscus analyzer (M Dialysis AB).

### Immunoassays

In-house enzyme-linked immunosorbent assays (ELISA) were used to measure C3a [31] and fluid-phase C5b-9 (sC5b-9) [32] concentrations in EDTA perfusate, urine samples and whole protein tissue extracts. Commercially available porcine ELISA assays were used to detect interleukin (IL)-10 (e-bioscience, Waltham, MA), tumor necrosis factor (TNF), IL-6 and IL-1β (R&D, Minneapolis, MN) in whole protein tissue extracts and EDTA perfusate. IL-8 quantification was performed using a Luminex assay (Merck, Darmstadt, Germany). All assays were used according to the manufacturer’s instructions. Tissue extraction was performed as previously described [33], using CytoBuster protein extraction reagent (EMD Millipore Corp., Billerica, MA) and cOmplete protease inhibitor cocktail (Roche, Basel, Switzerland).

### Kidney Damage Biomarkers and Function

Neutrophil gelatinase-associated lipocaline (NGAL) levels were detected by a commercially available porcine ELISA (Abcam, Cambridge, UK) according to the manufacturer´s instructions. Perfusate concentrations of creatinine were obtained through arterial blood gas, while the concentration in urine was measured using routine procedures at the clinical chemistry laboratory, Oslo University Hospital. Total protein concentrations in urine were measured by detergent compatible protein assay of Bio-Rad (Hercules, CA). Formulas used to estimate creatinine clearance and oxygen consumption are available in the Supplementary Material.

### Histological Evaluation

Histopathological injury was examined using hematoxylin & eosin and periodic acid-Schiff (PAS) staining techniques on paraffin-embedded biopsies. Glomerular capillary microthrombi and fibrin deposition were examined through a Maurits, Scarlet, and Blue (MSB) stain, as described in detail elsewhere [34]. Loss of glomerular integrity was scored on a scale of 0–100; 0 (none), 0–1 (occasional), 1–10 (mild), 10–50 (moderate) and severe (>50), the abundance of tubular protein casts was scored on an ordinal scale. Signs of tubular ischemic injury including intratubular cellular detachment and tubular necrosis were observed, but not quantified due to concerns raised about the accuracy of such subjective measurements in our setup. All histological analyses were performed by an experienced pathologist blinded to group allocation.

### Study Design and Statistical Analysis

In this prospective, blinded, controlled randomized study, kidneys were allocated randomly into two intervention groups using the random allocation tool in Microsoft Excel, the investigators handling the kidneys were blinded to the intervention. The sample size was calculated by power analyses, revealing that 10 kidneys in each treatment group would be sufficient to detect a 20% difference in the inflammatory markers (sC5b-9, TNF-α, IL-1β, IL-6, and IL-8) between the groups with a power of 0.8. In total, twenty-eight kidneys were included (sham, n = 6), two kidneys were excluded from analyses; one due to a technical perfusion defect and one due to morphologic abnormalities in the renal artery. NMP was terminated early when blood flow dropped below 10% of the maximum flow or severe perfusate leakage occurred, which was not possible to resuscitate within 5 minutes and/or kidney perfusion ceased. Six kidneys ceased functioning during NMP, in which five belonged to the C5-inhibitor treated group. One kidney ceased functioning after 74 min and one after 150 min and these were therefore excluded from analyses later than 60 min and 120 min of NMP, respectively. Four kidneys ceased functioning between 180 and 198 min of NMP and were excluded from analyses later than 180 min of NMP. Kidneys with perfusion times of ≥220 min were included in 240 min analyses. Values are presented as median ± interquartile range (IQR). Differences between C5 inhibitor-treated and control animals as well as differences over time throughout the study period were investigated using generalized linear mix model analyses (intervention as fixed effect and subject number as random effect). Non-parametric tests i.e., Mann-Whitney *U* test and Wilcoxon signed-rank test were used to compare differences between the groups. All statistical analyses were conducted using IBM SPSS Statistics for Macintosh 28 (IBM Cooperation, Armonk, NY) and GraphPad Prism 9 (GraphPad Software, San Diego, CA). P values less than 0.05 were considered statistically significant.

## Results

### Perfusion Characteristics During NMP

**FIGURE 2:**
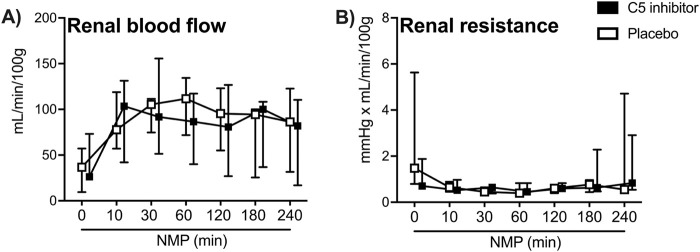
Perfusion characteristics. Arterial renal blood flow **(A)** and renal resistance **(B)** over a 240 min period of normothermic machine perfusion. Data are presented as median ± IQR. General mixed model analyses.

Kidney weight did not differ between control and the C5 inhibitor group at baseline (109 g versus 108 g, *p* = 0.784) or after NMP (150 g versus 151 g, *p* = 0.720). Perfusate characteristics were comparable between groups throughout the NMP period (Table 1). Mean arterial pressure was kept stable during NMP. The renal blood flow showed a steep increase during the initial 30 min and was stable thereafter until the end of the 4 h of NMP, with no difference between the control and C5-inhibitor treated group (*p* = 0.849, Figure 2A). The renal resistance decreased within the first 10 min and remained continuously low throughout the perfusion with no difference between the control and C5-inhibitor treated group (*p* = 0.282, Figure 2B).

**TABLE 1:** Perfusion solution characteristics during machine perfusion.

	*C5 inhibitor*				*Placebo*			
	T60	T120	T180	T240	T60	T120	T180	T240
Blood gas analysis								
pH	7.2 (7.1–7.3)	7.1 (7.1–7.3)	7.1 (6.9–7.2)	7.2 (7.0–7.2)	7.2 (7.2–7.2)	7.2 (7.1–7.3)	7.1 (7.0–7.2)	7.0 (6.9–7.1)
pO2 (kPa)	16.5 (11.8–17.1)	16.0 (14.8–17.0)	16.6 (13.7–17.8)	16.8 (15.0–18.5)	12.3 (9.2–15.0)	15.3 (13.3–16.3)	15.0 (13.6–17.8)	15.9 (14.0–18.7)
pCO2 (kPa)	3.5 (2.3–5.0)	2.9 (2.5–3.8)	3.2 (2.1–4.1)	2.8 (2.4–3.4)	3.8 (3.1–5.4)	4.1 (3.0–4.5)	4.3 (3.4–4.8)	2.8 (2.3–3.1)
Hb (g/dL)	6.6 (5.0–8.2)	6.3 (5.2–7.5)	6.0 (4.6–6.7)	8.2 (6.0–10.7)	7.7 (7.0–8.0)	6.7 (5.5–7.0)	6.4 (5.7–8.5)	6.9 (4.7–7.3)
Glucose (mmol/L)	4.1 (2.5–4.7)	6.1 (3.1–8.9)	3.7 (2.3–7.3)	7.1 (3.3–9.4)	4.5 (2.9–5.8)	5.7 (3.4–6.7)	5.7 (1.9–9.6)	6.0 (4.1–10.8)
Normothermic machine perfusion								
MAP	59.8 (53.3–63.2)	60.7 (56.3–62.3)	60.7 (55.0–65.0)	65.0 (62.3–67.6)	62.0 (58.7–64.0)	63.3 (58.0–69.5)	63.0 (60.6–64.3)	64.3 (60.6–69.0)

Abbreviations: MAP, mean arterial pressure.

### Renal Function and Injury

**FIGURE 3:**
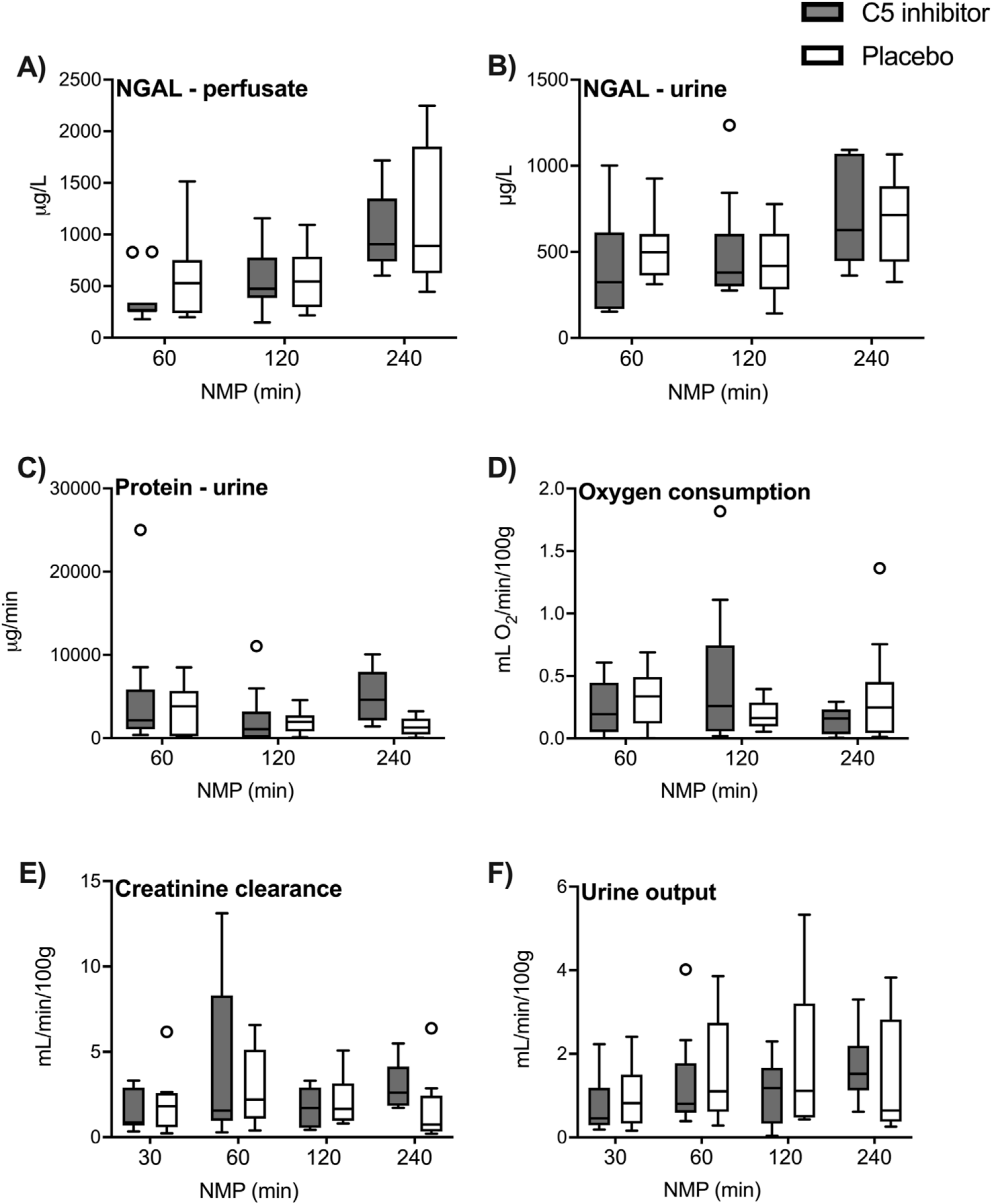
Renal function and injury. The renal function and injury markers in the control and the C5 inhibited group were compared over a 240 min period of normothermic machine perfusion. NGAL levels in perfusate and NGAL levels in urine **(A, B)**, excretion rates of protein in urine **(C)** and oxygen consumption creatinine clearance and urine production **(D–F)**. Data are presented as median ± IQR. Generalized mixed model analyses. NGAL, neutrophil gelatinase-associated lipocalin; NMP, normothermic machine perfusion.

Perfusate and urinary NGAL excretion rates significantly increased after 60 min (*p* = 0.001; *p* < 0.001, respectively Figures 3A, B). Throughout reperfusion, significantly higher levels of proteinuria were observed during the first hour of reperfusion (p = 0.032, Figure 3C). Generally low oxygen consumption levels were observed during NMP and plateaued at 0.20 (0.12–0.51) mL O_2_/min/100 g (Figure 3D). The creatinine clearance plateaued after 120 min NMP at 1.85 (0.94–2.98) mL/min/100 g (Figure 3E). Urine production rates slowly increased over the 4 h reperfusion period (∆ 0.72 mL/min/100g, *p* < 0.001, Figure 3F). No significant differences were observed between the control and the C5 inhibitor groups for assessed kidney function and injury markers.

### Renal Local Metabolism Assessed by Microdialysis

**FIGURE 4:**
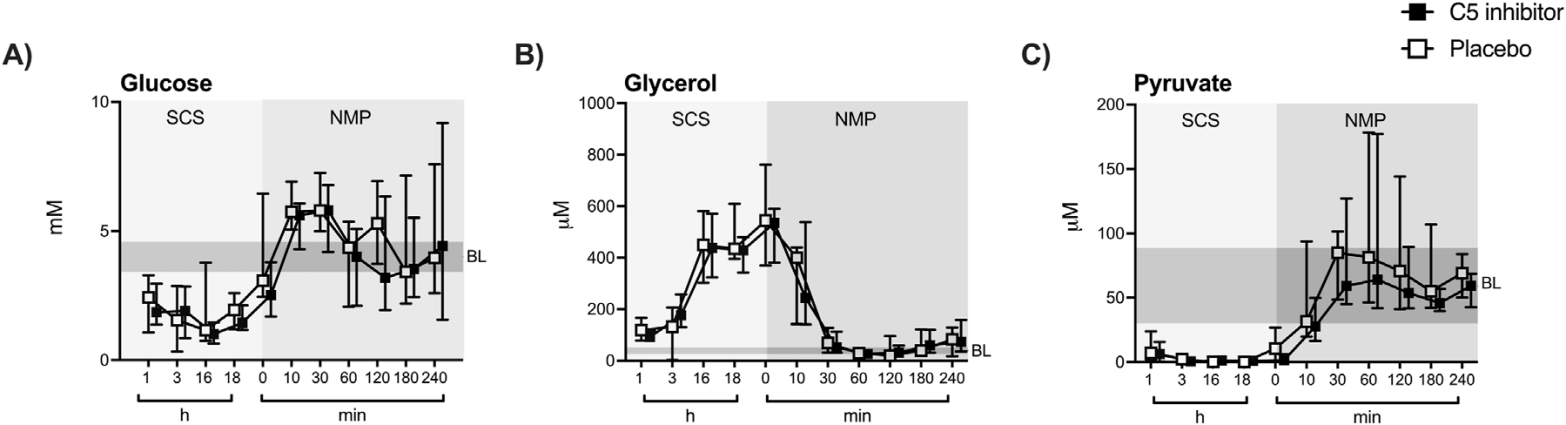
Renal tissue metabolism. Glucose, pyruvate and glycerol **(A–C)** were measured in the renal microdialysate during SCS and NMP. Data are presented as median ± IQR. General mixed model analyses. SCS, static cold storage; NMP, normothermic machine perfusion; BL, *in vivo* baseline measurements (mean ± 2x sd).

SCS led to a significant decline in tissue microdialysate levels of glucose (∆ −2.05 mM, *p* < 0.001) and pyruvate (∆ −56.82 µM, *p* < 0.001) Figures 4A, C), whereas glycerol levels increased (∆ +427.8 μM, *p* < 0.001) (Figure 4B) compared to *in vivo* baseline levels assessed prior to kidney procurement. A significant increase in glucose (∆ +3.81 mM, *p* < 0.001) and pyruvate (∆ +84.18 µM, *p* < 0.001) levels were observed during the initial 30 min of NMP, while glycerol levels decreased (∆ −378.8 μM, *p* < 0.001). Lactate was reduced upon SCS (∆ −1.91 mM, *p* < 0.001) and increased gradually during NMP (∆ +5.66 mM, *p* < 0.001) in comparison to baseline levels (Supplementary Figure S2). After 4 h of NMP, all metabolites settled at levels comparable to *in vivo* baseline levels except lactate, which showed a steady increase (Supplementary Figure S2). No statistical differences in microdialysis assessed metabolites were observed between the control and the C5 inhibitor group during SCS or NMP. Throughout NMP, correlations were observed between the level of tissue microdialysate levels of glycerol and urinary NGAL excretion rates (p = 0.0324, r = 0.316). None of the other kidney functional markers showed significant correlations (data not shown).

### Complement System Activation

**FIGURE 5:**
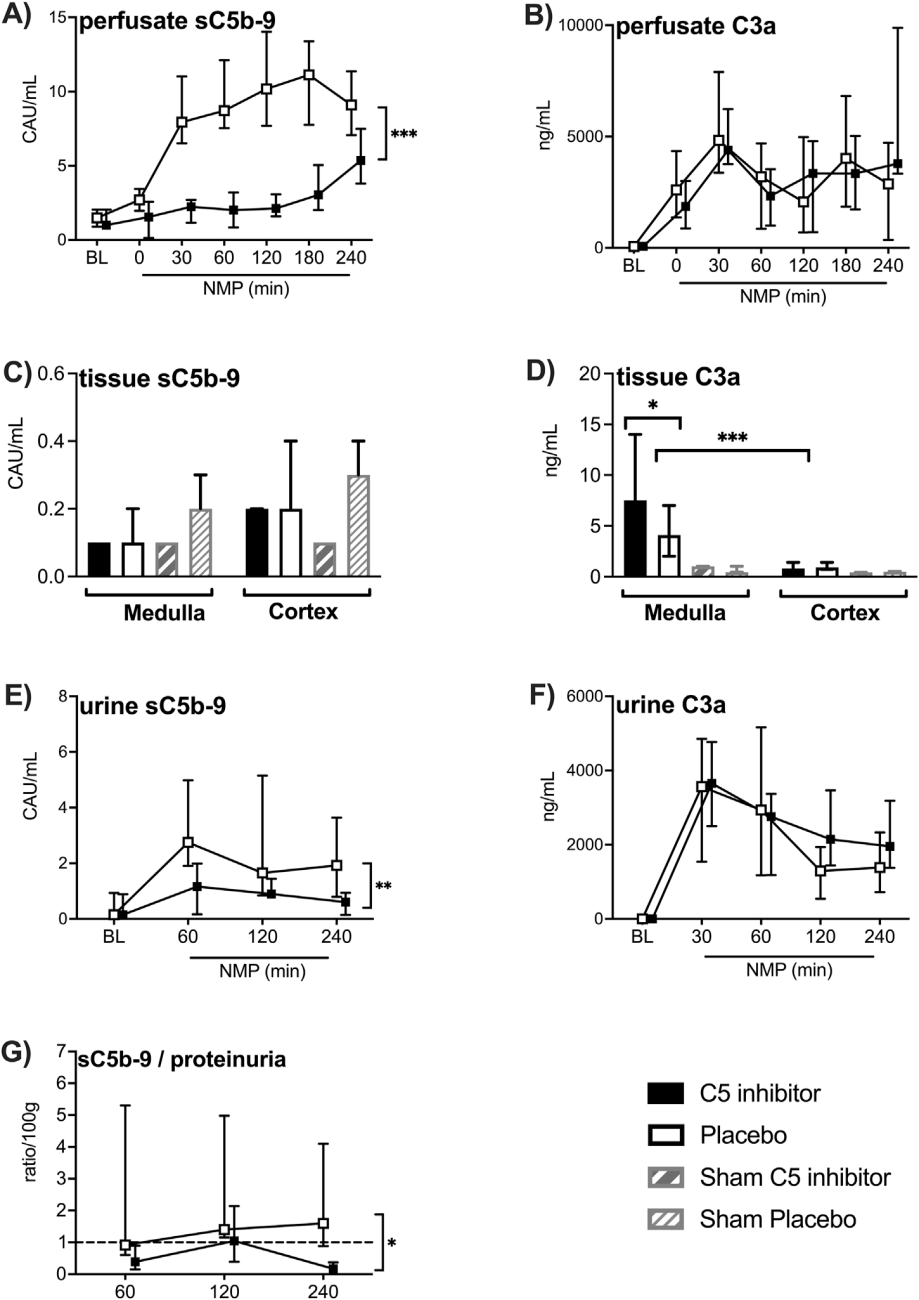
Effect of C5 complement inhibition on complement activation. The complement markers in the control and the C5 inhibited group were compared during and after a 240 min period of normothermic machine perfusion. sC5b-9 levels and C3a levels in the perfusate **(A, B)**, sC5b-9 and C3a levels in medulla and cortex tissue **(C, D)**, sC5b-9 and C3a levels in the urine **(E, F)** and urine sC5b-9-to-proteinuria ratio **(G)**. Data are presented as median ± IQR. General mixed model analyses, Wilcoxon signed rank test and Mann-Whitney-U test. * = p < 0.05, ** = p < 0.01, *** = p < 0.001. BL, *in vivo* baseline measurements; CAU, complement arbitrary units; NMP, normothermic machine perfusion.

C3a and sC5b-9 levels in the perfusate and urine increased during the initial 30 min of NMP and remained elevated for up to 4 h (Figures 5A, B, E, F). Over the whole NMP period, C5 inhibition led to significantly reduced levels of sC5b-9 in perfusate and urine (*p* < 0.001; *p* = 0.002, respectively), except for a modest but significant increase at 240 min NMP in perfusate compared to the start of NMP (*p* < 0.001). Lower urine sC5b-9-to-proteinuria ratios were observed in C5 inhibitor treated kidneys compared to non-treated kidneys (*p* = 0.033, Figure 5G). In contrast, C3a perfusate and urine levels did not differ between the control and the C5-inhibitor treated group according to inhibition at the C5 level (Figures 5B, F). No significant differences were observed in sC5b-9 from tissue extracts between the control and the C5 inhibitor group (Figure 5C). C3a tissue levels were significantly elevated after 4 h of NMP compared with sham-treated kidneys (*p* < 0.001) and were significantly higher in medulla tissue compared to cortex tissue (Figure 5D). In the medulla tissue, C5 inhibitor treated kidneys had significantly higher C3a tissue levels (*p* = 0.03) compared to placebo.

### Cytokine Production and Release

**FIGURE 6:**
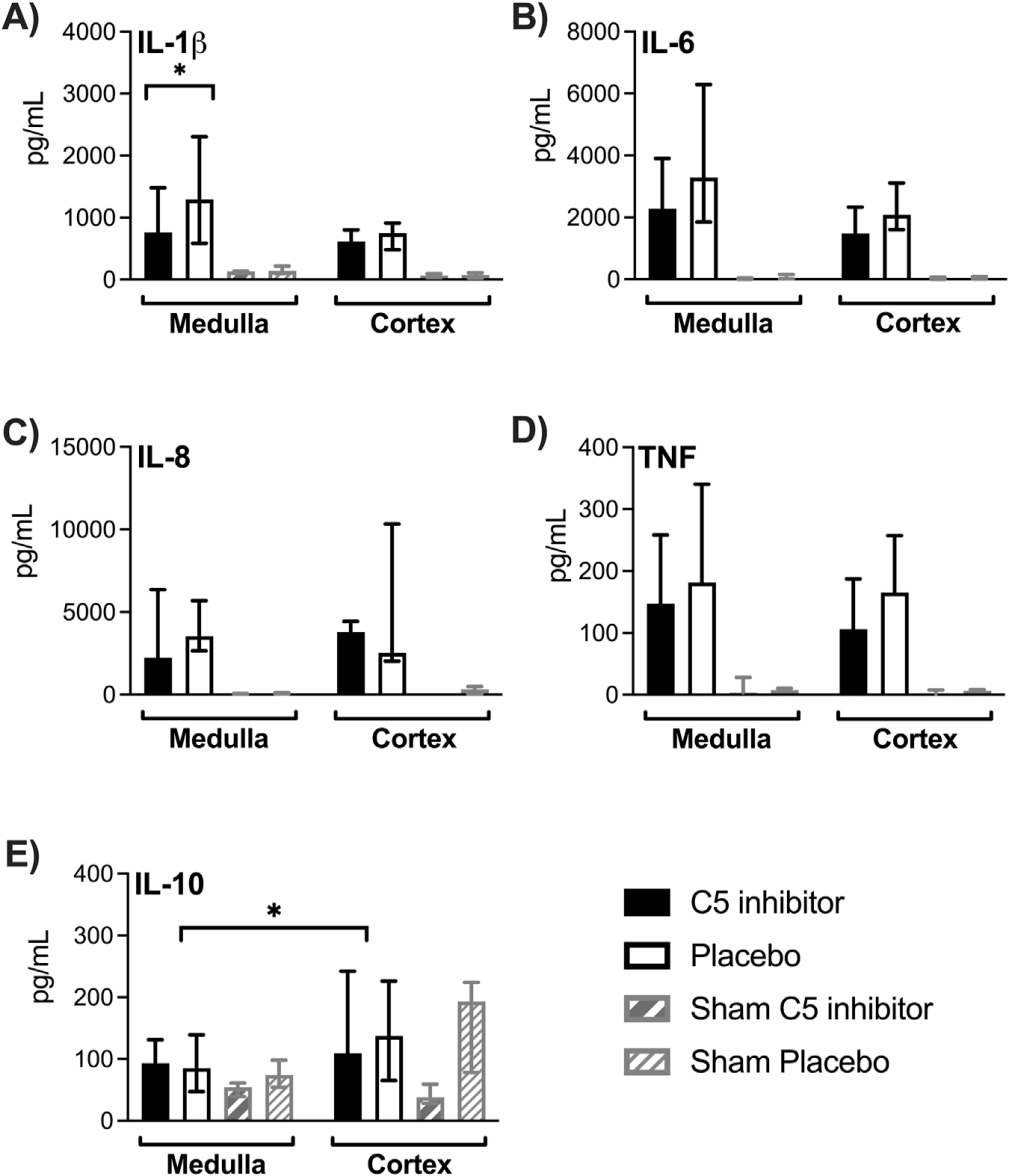
Effect of C5 complement inhibition on cytokine levels in renal tissue. The complement markers in the control and the C5 inhibited group were compared after a 240 min period of normothermic machine perfusion. IL-1β, IL-6, IL-8, TNF and IL-10 cytokine concentrations measured in medulla and cortex tissue **(A–E)**. Data are presented as median ± IQR. Wilcoxon signed rank test and Mann-Whitney-U test. * = p < 0.05. IL, interleukin; TNF, tumor necrosis factor.

All tissue cytokine concentrations, except IL-10, were significantly elevated after NMP compared to sham kidneys (*p* < 0.001 for all, Figures 6A–D). Cytokine concentrations did not significantly differ between medulla and cortex region, except for IL-10, which showed lower levels in medulla compared to cortex (*p* = 0.021, Figure 6E). C5 inhibitor treatment led to a 46% reduction of IL-1β levels in medulla tissue (*p* = 0.049), while only non-significant trends were observed for the other cytokines. Perfusate levels of IL-1β, IL-6, IL-8, TNF and IL-10 significantly increased after 120 min of NMP and remained elevated up to 4 h; no differences were observed between the control and the C5 inhibitor group (Supplementary Figure S3).

### Histopathology

**FIGURE 7:**
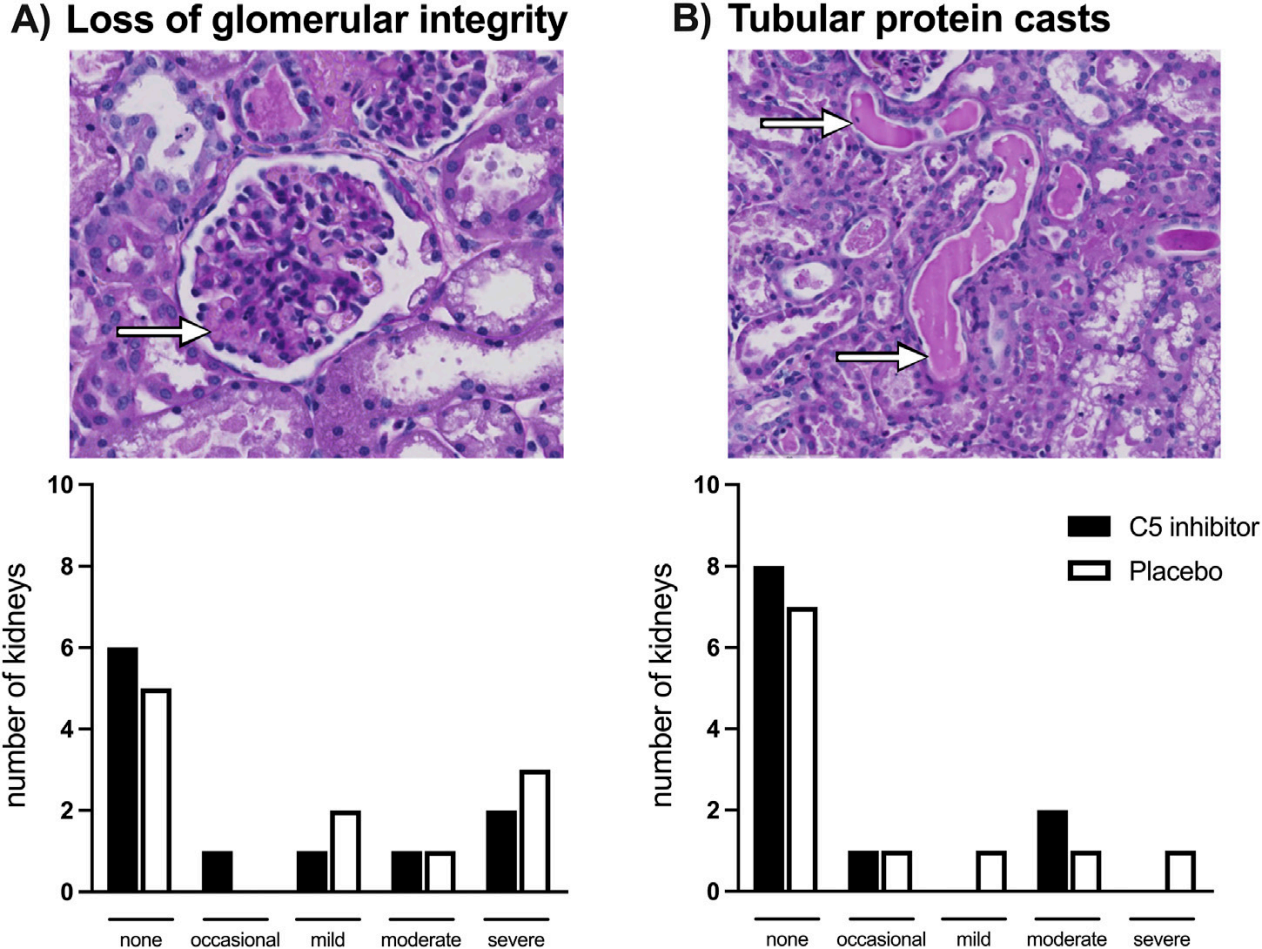
Histology. Loss of glomerular basement membrane integrity depicted by the arrow **(A)** and protein casts in the lumen of tubules depicted by the arrows **(B)** assessed after a 240 min period of normothermic machine perfusion in PAS-stained biopsies. No differences were observed between the control and the C5 inhibited group.

Glomerular basement membrane integrity loss together with a reduction in cell density of the mesangium was observed in several of the kidneys exposed to NMP; no differences were observed between the groups (Figure 7). In separate analyses, MSB staining showed no signs of intracapillary fibrin deposition. Protein casts were observed in the lumen of the tubules; most prominent in the calyces, without differences observed between the control and the C5 inhibitor group. No evident lesions were present among the sham-treated kidneys.

## Discussion

In this study, we demonstrated that microdialysis detected changes in the renal metabolites glucose, pyruvate and glycerol, comparable between both intervention groups in response to SCS and NMP. The primary aim of this study was to assess the effect of complement inhibition on NMP induced inflammatory responses. We observed that NMP induced inflammation with increase in complement and cytokine levels in perfusate, urine, and kidney tissue. C5 inhibition completely blocked sC5b-9 formation and substantially and significantly reduced IL1-β, a central component of the NLRP3 inflammasome.

During organ transplantation, the metabolic state of kidney grafts is affected and NMP is used to reconstitute metabolism with the aim to reduce organ damage upon reperfusion [14]. Here, SCS caused a decrease in glucose and pyruvate levels while glycerol increased. These findings are consistent with previous studies and confirm the lack of metabolic function during SCS and cellular membrane break-down reflected by glycerol increase [18, 35, 36]. Upon the initiation of NMP, microdialysis detected an immediate glucose increase, followed by pyruvate, whereas the level of glycerol dropped significantly. Thus, NMP leads to return of renal cellular metabolism and decreases fatty-acid breakdown. Lactate increased progressively during NMP. Similar metabolic trends have also been found in the perfusate of previous NMP studies [12, 37–39] and are due to the limited ability of the kidney to metabolize lactate. The accumulation of lactate might be explained by the release through activated erythrocytes and leucocytes present in the perfusate [40, 41]. Renal lactate production caused by reduced oxidative phosphorylation [42] is less likely since pyruvate stabilized at prior *in vivo* levels. Renal oxygen consumption was stable. Thus, lactate was not produced by renal but leucocyte hypermetabolism.

NMP led to a significant activation of the complement system as revealed by increase in the activation products C3a and sC5b-9 in perfusate, urine, and renal tissue. These findings are consistent with studies assessing complement activation in other extracorporeal blood circulations such as cardiopulmonary bypass, hemodialysis and plasmapheresis [25] and in-line with previous findings in pig and human kidney NMP from our group [43]. The introduction of foreign material or a gas-blood interface into the circulation could initiate complement activation [44]. Artificial and air surfaces have been shown to induce IgG and C3 conformational changes resulting in the activation of the classical and alternative pathways [44–46]. Here, we have minimized gas-plasma interfaces, by using a closed-NMP system. Local synthesis of complement proteins by the kidney itself could be an important contributor [47]. Human kidney NMP uses plasma-free perfusates, but still shows complement activation, which can be explained by small amounts of plasma left in the kidney as well as *de novo* synthesis of complement in the kidney [43]. Thus, although NMP reconstituted metabolism, a strong innate immune reaction was induced, which might hamper organ function and could at least in part explain high delayed graft function rates in clinical trials of kidney NMP after SCS preservation [6].

C5-inhibition blocked perfusate and urine sC5b-9 formation throughout NMP. sC5b-9 concentrations extracted from tissue were low and comparable between groups. Thus, we did not assess the deposition of C5b-9 in tissue sections. Furthermore, it is known that complement activation can induce endothelial cell and immune cell activation without detectable tissue complement activation [48]. Clinical trials evaluated the efficacy of the C5 inhibitor eculizumab when given minutes prior to reperfusion of kidney grafts and reported no benefit on delayed graft function [49]. In our study, kidney reperfusion was mimicked by using whole blood during NMP and our results are consistent with findings from these clinical trials. Here, C5 inhibition was extended and started immediately after organ procurement. However, C5 inhibition did not affect metabolic or physiological markers of kidney function, implying that transplant-induced IRI is only partly C5 dependent. Studies in mice imply that the lectin and alternative complement pathways contribute to renal IRI; mice deficient in MBL, factor B, or C3 showed reduced renal injury [50, 51]. Activation of these pathways results in the cleavage of C3 into C3a and C3b fragments. Since C3a-receptors are expressed on renal tubular epithelial cells and granulocytes, C3a is thought to play a role in the pathogenesis of renal IRI [28, 52]. In this study, C5 was inhibited and thus C3 cleavage led to similar C3a generation in both groups. Thus, targeting C3-cleavage might provide better outcomes. Unfortunately, there is no effective porcine C3 inhibitor currently available.

NMP caused a significant increase in the level of cytokines in the perfusate and tissue after 60–120 min from the start of NMP. Concordant with our findings, Stone *et al.* observed an inflammatory storm after kidney NMP, demonstrated by the increase of a range of pro-inflammatory cytokines at high concentrations [53], which has been confirmed in discarded human kidneys [43]. Interestingly, C5 inhibition resulted in a decrease of 46% in IL-1β levels in kidney tissue. Increased IL-1β levels have been linked with decreased graft function following IRI and co-occur in many diseases caused by complement dysregulation [50, 54]. We speculate that cytokines were induced by DAMPS originating from the initial oxidative allograft injury as the use of autologous blood only allows for stimulation by “self” molecules [13, 55]. In line with Jager *et al.*, TNF perfusate levels rapidly increased upon NMP whereas the other cytokines increased first after 1 hour [43]. This strengthens the notion that a TNF-dependent pathway might be involved in generation of cytokines [43]. The levels of cytokines decreased at the end of perfusion. A dilution effect is less likely as we observed steady hemoglobin concentrations, implying that the observed decrease reflects a biological mechanism. Taken together, all studied cytokines increased in our study after NMP. As cytokines are induced by several innate immune sensor systems and renal IRI has been shown to enhance both TLR2 and TLR4 expression, the combined inhibition of complement with TLR co-factor CD14 may be more effective [56–58].

A limitation of this study is that we used whole blood as perfusate. Initial experiments had shown vast complement activation during leukocyte filtration of pig whole blood, which could have influenced the results [59]. However, also NMP performed with leucoyte- and even plasma-free perfusate has been reported to activate complement and cytokine production during NMP [43]. Thus, the results of this study might be useful also in clinical settings of kidney NMP. However, C5 inhibition in a clinical study [49] and this study did not lead to improvement in immediate kidney function and tissue damage. Thus, future research might have higher chances of success if optimization of NMP includes metabolic and inflammatory interventions in combination. A small number of kidneys were investigated in this study, but the paired approach using both kidneys from each individual created an ideal platform for assessing the C5 intervention. All kidneys returned function. Nonetheless, the observed histological injury along with enhanced NGAL levels and proteinuria confirmed renal IRI in our model. Some kidneys cease functioning due to high resistance before the 4-h endpoint. While up to 8 h kidney NMP is described, most NMPs are carried out in an open system [60]. Our closed-circuit setup required continuous monitoring to correct for volume loss and was highly susceptible to obstruction caused by collapse of the renal vein. Future research comparing the degree of complement activation in both systems should provide an answer to whether there is a rationale for using the more labor-intensive closed-circuit system.

In conclusion, metabolism can be assessed by microdialysis in kidney NMP and reveals metabolic demands during NMP. NMP induced complement activation and production and release of cytokines. Renal inflammation upon IRI appeared to be partially mediated by the complement system as C5 inhibition mainly led to non-significant changes except for a marked and significant decrease of IL-1β. However, C5 inhibition did not lead to improvement of kidney function and tissue damage. Further metabolic optimization of the NMP model and the assessment of additional immune inhibitors, should be the next step to reduce NMP-induced renal IRI.

## Data Availability

The original contributions presented in the study are included in the article/Supplementary Material, further inquiries can be directed to the corresponding author.

## Abbreviations

DGF, delayed graft function; EDTA, ethylenediaminetetraacetic acid; ELISA, enzyme-linked immunosorbent assays; HMP, hypothermic machine perfusion; IL, interleukin; IQR, interquartile range; IRI, ischemia reperfusion injury; KPS, kidney preservation solution; MAP, mean arterial pressure; NGAL, neutrophil gelatinase-associated lipocaline; NMP, normothermic machine perfusion; PAS, periodic acid-schiff; SCS, static cold storage; sC5b-9, fluid-phase C5b-9; TNF, tumor necrosis factor.
