# Supplementary material for: Normothermic Machine Perfusion Reconstitutes Porcine Kidney Tissue Metabolism But Induces an Inflammatory Response, Which Is Reduced by Complement C5 Inhibition
Source: Transpl Int. 2024 Nov 13;37:13348. doi: 10.3389/ti.2024.13348 (PMC11598510; doi:10.3389/ti.2024.13348)
Supplement: Supplementary file 1 [file DataSheet1.pdf]

Supplemental Material

<u>Material and methods</u>

Oxygen consumption (mL O_2/min/100g)

$$\left(\frac{((\mathrm{Hb\ x\ 2.4794}) + (\mathrm{pO_{2\ arterial}\ x\ K})) - ((0.02794\ \mathrm{x\ Hb\ x\ SO_{2\ venous}}) + (\mathrm{pO_{2\ venous}\ x\ K})\mathrm{x\ Q})}{\mathrm{g}}\right)\ \mathrm{x}\ 100$$

Hb = haemoglobin (mmol/L), pO_2 = perfusate partial pressure (mmHg), K = 0.0225 (mL O_2/kPa) solubility constant of oxygen in water at 37ºC, SO_2 =saturated oxygen (%), Q = renal blood flow (L/min), g = baseline kidney weight in grams.

Creatinine clearance (mL/min/100g)

$$\frac{\left(\frac{\mathrm{U_{creatinine}\ x\ urine\ volume}}{\mathrm{P_{creatinine}}}\right)}{\mathrm{g}}\ \mathrm{x}\ 100$$

Ucreatinine = creatinine concentration in the urine (mmol/L), urine volume = (mL/min), Pcreatinine = creatinine concentration in the perfusate (mmol/L), g = baseline kidney weight in grams.

Figure S1. Titration of the C5 inhibitor to select the optimal dose blocking sC5b-9 formation

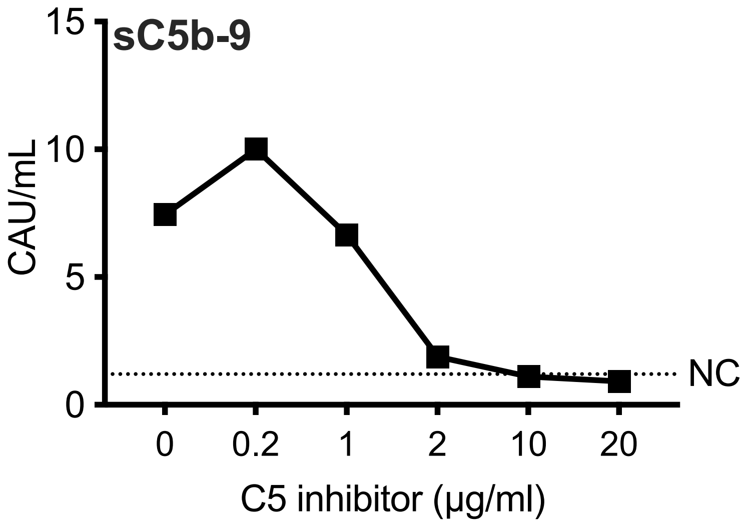

Dose-dependent inhibition of sC5b-9 formation by the C5 inhibitor in zymosan activated pig whole blood. CAU, complement arbitrary units; NC, negative control.

Figure S2. Lactate tissue metabolism

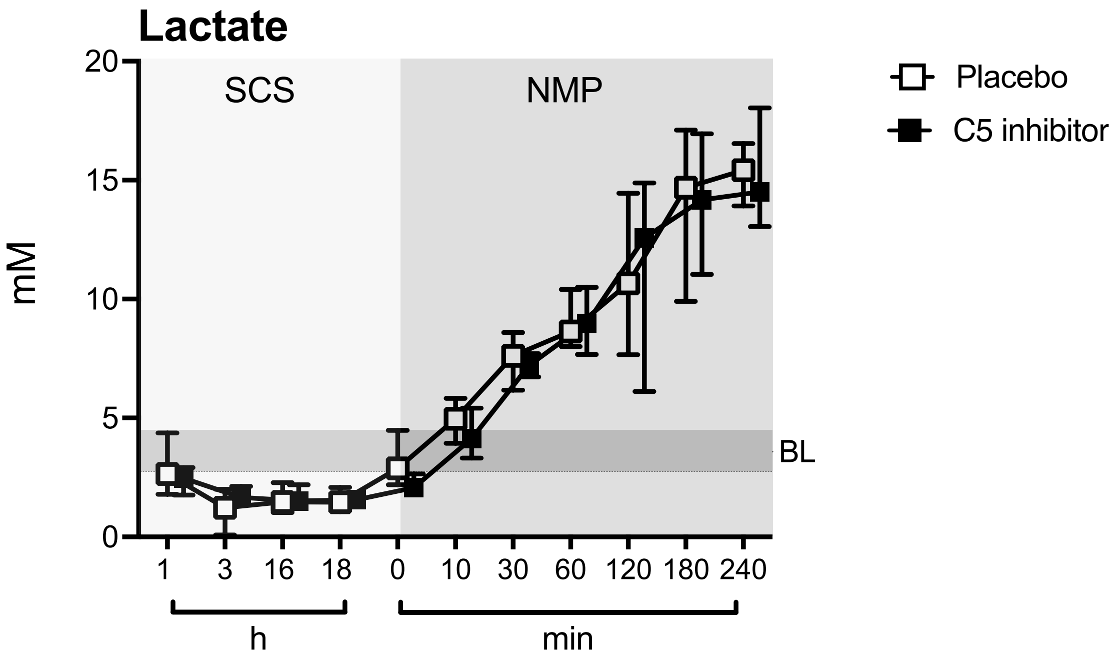

Lactate was measured in the renal microodialysate during SCS and NMP. Data are presented as median ± IQR. General mixed model analyses. SCS, static cold storage; NMP, normothermic machine perfusion; BL, *in vivo* baseline measurements (mean ± 2x sd).

Figure S3. Effect of C5 complement inhibition on cytokine levels in the NMP perfusate

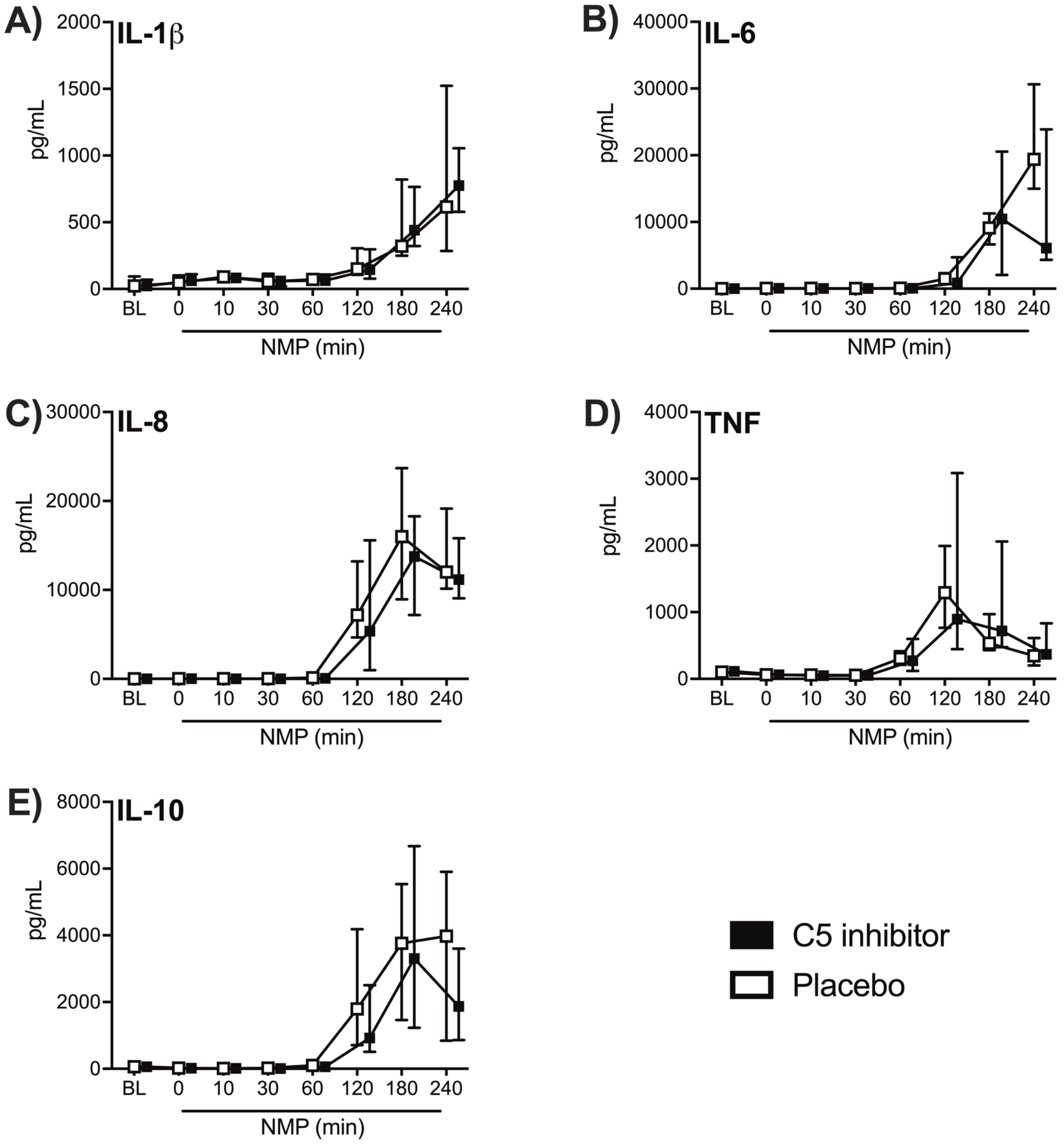

The perfusate IL-1β, IL-6, IL-8, TNF and IL-10 cytokine concentrations in the control and the C5 inhibited group were compared over a 240 min period of normothermic machine perfusion (A-E). Data are presented as median ± IQR. General mixed model analyses. BL, *in vivo* baseline measurements; NMP, normothermic machine perfusion.
